# Increased serum neurofilament light chain concentration indicates poor outcome in Guillain-Barré syndrome

**DOI:** 10.1186/s12974-020-01737-0

**Published:** 2020-03-17

**Authors:** Patrick Altmann, Desiree De Simoni, Alexandra Kaider, Birgit Ludwig, Jakob Rath, Fritz Leutmezer, Fritz Zimprich, Romana Hoeftberger, Michael P. Lunn, Amanda Heslegrave, Thomas Berger, Henrik Zetterberg, Paulus Stefan Rommer

**Affiliations:** 1 Department of Neurology, Medical University of Vienna, Vienna, Austria; 2 Division of Neuropathology and Neurochemistry, Medical University of Vienna, Vienna, Austria; 3 Center for Medical Statistics, Informatics and Intelligent Systems, Medical University of Vienna, Vienna, Austria; 4 Neuroimmunology and CSF Laboratory, Institute of Neurology, University College London, London, UK; 5 Department of Neurodegenerative Disease, UCL Queen Square Institute of Neurology, London, UK; 6 The UK Dementia Research Institute at UCL, London, UK; 7 Department of Psychiatry and Neurochemistry, The Sahlgrenska Academy at the University of Gothenburg, Institute of Neuroscience and Physiology, Mölndal, Sweden; 8 Clinical Neurochemistry Laboratory, Sahlgrenska University Hospital, Mölndal, Sweden

**Keywords:** Guillain-Barré syndrome, Biomarker, Neurofilament, Prognosis, Outcome

## Abstract

**Background:**

Guillain-Barré syndrome (GBS) is an autoimmune disease that results in demyelination and axonal damage. Five percent of patients die and 20% remain significantly disabled on recovery. Recovery is slow in most cases and eventual disability is difficult to predict, especially early in the disease. Blood or cerebrospinal fluid (CSF) biomarkers that could help identify patients at risk of poor outcome are required. We measured serum neurofilament light chain (sNfL) concentrations from blood taken upon admission and investigated a correlation between sNfL and clinical outcome.

**Methods:**

Baseline sNfL levels in 27 GBS patients were compared with a control group of 22 patients with diagnoses not suggestive of any axonal damage. Clinical outcome parameters for GBS patients included (i) the Hughes Functional Score (HFS) at admission, nadir, and discharge; (ii) the number of days hospitalised; and (iii) whether intensive care was necessary.

**Results:**

The median sNfL concentration in our GBS sample on admission was 85.5 pg/ml versus 9.1 pg/ml in controls. A twofold increase in sNfL concentration at baseline was associated with an HFS increase of 0.6 at nadir and reduced the likelihood of discharge with favourable outcome by a factor of almost three. Higher sNfL levels upon admission correlated well with hospitalisation time (*r*_*s*_ = 0.69, *p* < 0.0001), during which transfer to intensive care occurred more frequently at an odds ratio of 2.4. Patients with baseline sNfL levels below 85.5 pg/ml had a 93% chance of being discharged with an unimpaired walking ability.

**Conclusions:**

sNfL levels measured at hospital admission correlated with clinical outcome in GBS patients. These results represent amounts of acute axonal damage and reflect mechanisms resulting in disability in GBS. Thus, sNfL may serve as a convenient blood-borne biomarker to personalise patient care by identifying those at higher risk of poor outcome.

## Introduction

The prognosis in some cases of Guillain-Barré syndrome (GBS) is rather poor—a fact partly attributed to the challenge of identifying these patients early in their presentation. Despite immunotherapy, one in five remains severely disabled. The mortality rate is 5% and mainly driven by complications requiring transfer to an intensive care unit (ICU) [1–3]. The diagnosis of GBS remains clinical, yet it is interlaced with published criteria for GBS that encompass nerve conduction studies (NCS) and analyses of cerebrospinal fluid (CSF) [4, 5]. Treatment strategies rely on early diagnosis; plasma exchange shortens time to neurological improvement, with intravenous immunoglobulin being equivalent. The highest efficacy is achieved when treatment started within 2 weeks from disease onset [6–9]. Although recent research has put the complement system under the microscope, no significant alterations to prognosis were accomplished in the past 30 years [10, 11].

Outcome prediction in patients with GBS is difficult due to a substantial variation in phenotypes. A few outcome scores provide some early prognostic data based on clinical presentation [12, 13]. However, there are currently no early predictive fluid biomarkers available. Hence, there is an urgent need to discover reliable, valid, and easily measurable serum; plasma or urine biomarkers that would guide caregivers in their clinical decision endeavour.

Neurofilaments have aroused considerate attention in biomarker research in a variety of neurological disorders [14–17]. Cellular disruption results in release of axonal cytoskeletal protein family neurofilaments into the CSF and serum [18–20]. A few previous studies in small cohorts of GBS patients have demonstrated prognostic properties for neurofilament heavy chain (NfH) and light chain (NfL) levels [21–24].

In the present study, we used state-of-the-art single molecule array (Simoa) technology to determine serum NfL (sNfL) levels in both GBS patients and controls. We systematically studied hospital records of 27 patients with diagnoses of GBS and its variants and applied advanced statistical analyses to expose any possible associations of sNfL levels with GBS disease course. The results are discussed in terms of measured biomarker concentration upon hospital admission and outcome at discharge.

## Methods

This study was approved by the ethics committee at the Medical University of Vienna (EK1283/2018).

### Study population

**Fig. 1:**
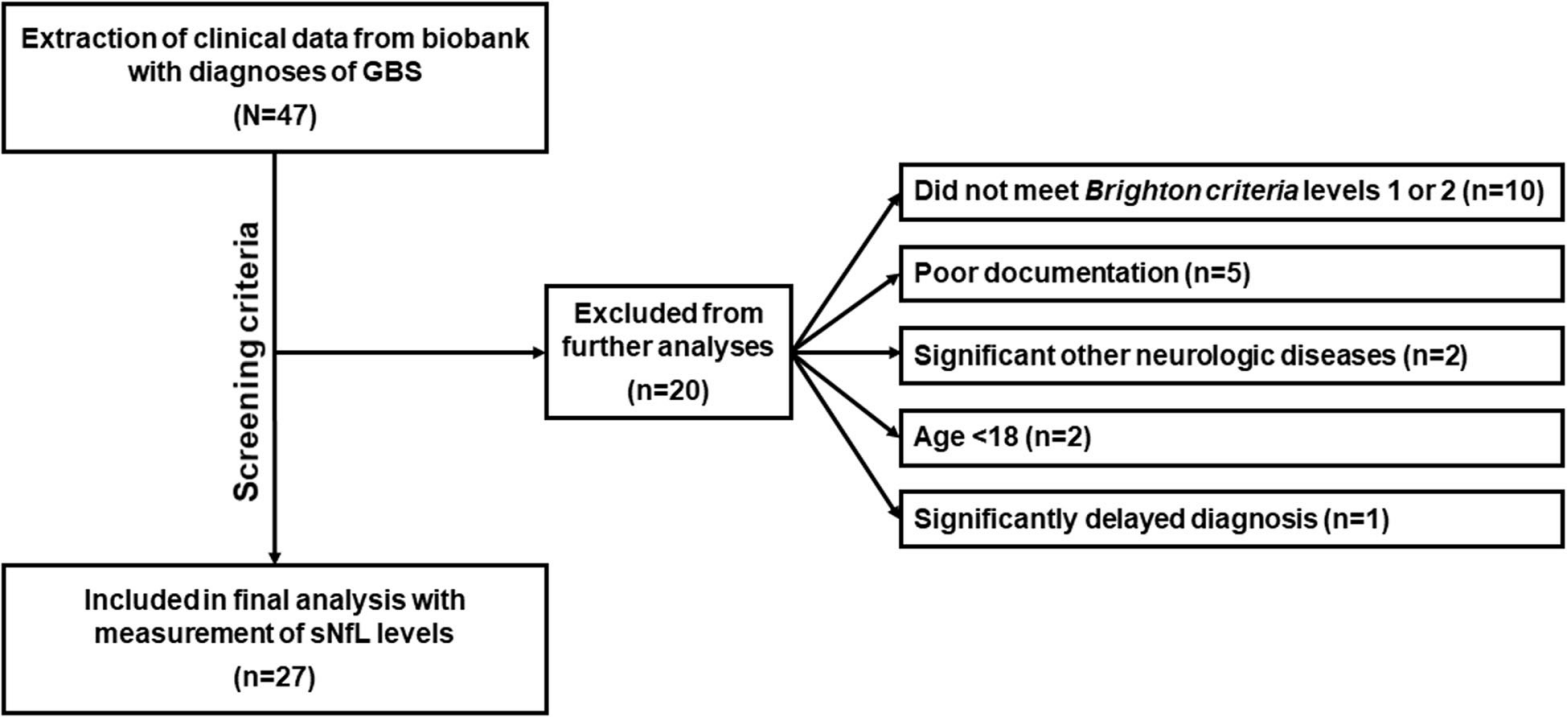
Flow chart of the selection process of GBS (Guillain-Barré syndrome) patients with the application of exclusion criteria

For this retrospective study, we screened our local biobank for suspected diagnoses of GBS or acute inflammatory demyelinating polyradiculoneuropathy (AIDP) or acute motor axonal neuropathy (AMAN) or acute motor and sensory axonal neuropathy (AMSAN) or Miller-Fisher syndrome from April 2014 through February 2018. A total of 47 patients were identified. Reviewing these patients’ clinical reports, 27 cases were included in the final study based on pre-defined screening criteria (Fig. 1). Screening criteria consisted of concise documentation of clinical disease course with the presence of detailed reports for nerve conduction studies (NCS); documented neurological exams; CSF results and biobank blood obtained within 5 days of hospitalisation; no previous diagnosis of GBS or preceding treatment in another facility; age > 18; and no documented comorbidities that would be indicative of altered sNfL levels (such as multiple sclerosis, any neurodegenerative disorder, stroke, seizure disorders, brain and spinal cord trauma, brain and spinal cord tumours, any neuropathy, congenital neurological disorders). Finally, only patients meeting levels 1 or 2 of the Brighton criteria were included [25]. Serum samples from 22 patients without any known axonal damage (benign intracranial hypertension and psychiatric disorders excluding dementia) served as a control group.

### Extraction of clinical and paraclinical data

We extracted the following parameters from the electronic patient documentation system: (i) the Hughes Functional Score (HFS), a neurological evaluation score on general functioning in GBS patients, ranging from 0 (normal health), 1 (minor neurological symptoms or signs, being able to run), 2 (able to walk at least 5 m, but unable to run), 3 (able to walk 5 m with walker or support), 4 (bedridden), 5 (ventilated), to 6 (dead) [8]. HFS was calculated at admission (HFS*a), nadir (HFS*n) and discharge (HFS*d); (ii) detailed results from NCS with classification according to the Hadden criteria (that is primary demyelinating, primary axonal, inexcitable, equivocal or normal) [4]. These electrophysiology reports and the classification of GBS (and its variants) were confirmed by an investigator blinded to the clinical data based on standard nerve conduction studies of motor nerves including measurement of distal motor latency, CMAP amplitude and proximal/distal CMAP amplitude ratio, motor conduction velocity and minimal F wave latency; (iii) important results from the CSF analysis (cell count, total protein levels); (iv) evidence whether GBS onset was associated with a preceding infection (upper respiratory tract infection or diarrhoea); (v) information on treatment strategies and (vi) results from testing for anti-ganglioside antibodies.

### Blood sampling

Serum samples were obtained within 5 days after symptoms of sensorimotor weakness occurred. Peripheral venous blood was collected in Greiner Bio-One Vacuette serum tubes (GBO, Kremsmuenster, Austria) and sent to the local biobank where the blood was processed according to standard operating procedures in an ISO 9001-certified environment as described previously [26]. In brief, tubes were centrifuged at 1.884×*g* for 10 min at room temperature after clotting had completed. Serum was then transferred to virgin polypropylene tubes in 400 μL aliquots and subsequently stored at − 70 °C until analysis.

### Analysis of serum neurofilament light chain concentrations

For NfL measures, samples were thawed for 60 min at room temperature and were analysed by an investigator blinded to clinical data using the Simoa Nf-light kit in the Simoa HD-1 Analyser (Quanterix, Lexington, MA, USA), which runs ultrasensitive paramagnetic bead-based enzyme-linked immunosorbent assays [27]. For this protocol, briefly, thawed samples and calibrators were dispensed in provided 96-well plates as duplicates. Further sample processing (dilution, incubation, washing, shaking, resuspending and reading) was carried out in an automated manner as described elsewhere [28]. The sNfL assay was carried out according to the manufacturer’s instructions and protocol.

### Statistical analyses

Continuous variables are described by the mean (±SD) or the median value (interquartile range, IQR) in case of non-normal distributions. Due to the skewed distribution of sNfL, we used log2-transformed values for statistical analyses. To investigate differences in sNfL levels in GBS patients compared with controls, the unpaired *t* test was used, and analyses of covariance were applied to adjust the comparison for the possible confounder age and sex. To describe the association between sNfL and age, the Pearson correlation coefficient (*r*_*p*_) was calculated. The association of sNfL, with CSF protein, albumin index, HFS and duration of hospitalisation, is described by the Spearman correlation coefficient (*r*_*s*_). The duration of hospitalisation was set to the maximum value for two patients who had died at the ICU. Separate univariate and multivariable linear regression models were performed to evaluate the influence of sNfL on HFS*a and HFS*n, respectively. The HFS*a (HFS*n) value was considered as dependent variable and sNFL (log2 transformed) as independent explanatory variable. In the multivariable models, age (in years) and detection of a preceding infection (yes vs. no) were additionally included as explanatory variables. Since 74% of the patients in our cohort had HFS*d values = 1, the binary outcome variable HFS*d = 1 vs. ≥ 2 was analysed using univariate logistic regression analyses. No adjustment was possible due to the small number of patients with HFS*d values ≥ 2. Univariate and multivariable Cox regression models were used to evaluate the influence of sNfL on the duration of hospitalisation, where two patients who had died at the ICU were censored at a maximum value. Age and preceding infection were included in addition to sNfL as explanatory variables in the multivariable regression model. Furthermore, univariate logistic regression model was performed to evaluate the prognostic value of sNfL on the probability of a transfer to an ICU, including receiver operating characteristic (ROC) curve analyses. Two-sided *p* values < 0.05 were considered statistically significant. The software used was the SAS version 9.4 (SAS Institute Inc. (2016), Cary, NC, USA).

## Results

### Clinical history and nerve conduction were compatible with the diagnosis of GBS

All 27 patients fulfilled levels 1 or 2 of the Brighton diagnostic criteria regarding their clinical presentation: bilateral and flaccid weakness of the limbs, decreased or absent deep tendon reflexes, monophasic course and no alternative explanation for their symptoms. Out of these GBS patients, preceding infection was reported in 18/27 patients. As for the electrophysiological GBS classification, NCS reports from all 27 patients were available and compatible with diagnoses of GBS. All NCS reports were re-evaluated according to current diagnostic criteria. Nine reports showed missing single values and could not be re-verified, as machines were updated and protocols changed. Yet still, they all confirmed the diagnosis of GBS. Demographic information, results from nerve electrophysiology, sNfL levels on admission, CSF results, information on treatment strategies and results from anti-ganglioside antibody testing are given in Table 1. Neurophysiological features in our samples were heterogeneous. Seventeen patients showed a primary demyelinating pattern, five patients exhibited primary axonal changes and five patients had equivocal abnormalities. Due to small sample size across these three groups, no statistical calculations were possible. We found positive test results for antiganglioside antibodies in three non-demyelinating variants of GBS. Based on their clinical presentation, results from NCS and antiganglioside antibodies, we classified all 17 patients with primary demyelinating features as AIDP and two patients with primary axonal abnormalities on NCS as AMAN and one as AMSAN.

**Table 1:** Patient characteristics

Patient no.	Sex	Age	sNfL (pg/ml)	CSF dissoc.	CSF protein	Albumin index	NCS	Diagnostic certainty	Treatment	Ganglioside
1	**m**	**53**	**88.9**	No	34.1	4.2	Primary demyelinating	2	IVIg	Neg
2	**f**	**80**	**246.4**	Yes	119.8	23.2	Primary demyelinating	1	IVIg	Neg
3	**m**	**48**	**238.0**	Yes	54.8	12.6	Primary demyelinating	2	IVIg, PE, ICU	Neg
4	**m**	**65**	**65.0**	No	44.3	5.5	Primary demyelinating	2	IVIg	Neg
5	**m**	**77**	**57.8**	No	43.4	5.8	Primary demyelinating	2	IVIg, PE, ICU	Neg
6	**f**	**59**	**45.0**	Yes	68.8	12.6	Primary demyelinating	1	IVIg	Neg
7	**f**	**74**	**135.3**	Yes	175.6	35.0	Primary demyelinating	1	IVIg, ICU	Neg
8	**f**	**45**	**71.7**	No	36.0	5.2	Primary demyelinating	2	IVIg	Neg
9	**m**	**52**	**77.3**	Yes	57.2	12.9	Primary demyelinating	1	IVIg	Neg
10	**m**	**65**	**17.9**	Yes	53.2	8.9	Primary demyelinating	1	IVIg	Neg
11	**m**	**37**	**383.0**	Yes	67.5	17.8	Primary demyelinating	1	IVIg, PE	Neg
12	**m**	**20**	**229.7**	Yes	76.7	12.9	Primary demyelinating	1	IVIg, PE, ICU	Neg
13	**m**	**54**	**204.5**	No	47.4	17.3	Primary demyelinating	1	IVIg, PE, ICU	Neg
14	**m**	**70**	**41.5**	No	45.1	5.3	Primary demyelinating	2	IVIg	Neg
15	**m**	**74**	**249.0**	Yes	55.2	15.8	Primary demyelinating	1	IVIg	Neg
16	**f**	**22**	**499.8**	Yes	157.8	34.9	Primary demyelinating	1	IVIg, PE, ICU	Neg
17	**m**	**73**	**146.6**	Yes	70.5	14.0	Primary demyelinating	1	IVIg	Neg
18	**w**	**30**	**30.7**	Yes	16.7	2.3	Primary axonal	2	IVIg	GM1
19	**f**	**78**	**12.9**	No	45.3	6.0	Primary axonal	1	IVIg	Neg
20	**m**	**66**	**85.5**	Yes	102.5	21.0	Primary axonal	1	IVIg	Neg
21	**f**	**66**	**46.7**	No	28.5	4.6	Primary axonal	2	IVIg, PE, ICU	GM1
22	**m**	**30**	**139.0**	No	32.1	4.6	Primary axonal	2	IVIg, PE, ICU	GD1a
23	**m**	**31**	**17.2**	Yes	86.5	14.0	Equivocal	1	IVIg	Neg
24	**f**	**40**	**82.6**	Yes	74.2	13.6	Equivocal	1	IVIg	Neg
25	**f**	**34**	**18.6**	Yes	30.2	4.1	Equivocal	1	IVIg	Neg
26	**m**	**74**	**210.5**	No	46.8	9.7	Equivocal	1	IVIg, ICU	Neg
27	**w**	**59**	**684.7**	Yes	76.4	13.0	Equivocal	1	IVIg, PE, ICU	Neg

Analysed data from this study cohort (*n* = 27): demographic information, serum neurofilament light chain (sNfL) concentration upon admission, CSF results (albumin-cytological dissociation, total CSF protein count [mg/dl] and albumin index [× 1000]), results from nerve conduction studies (NCS) according to *Hadden* criteria [4], the level of diagnostic certainty (Brighton criteria [25]), information on the treatment strategy (*IVIg* intravenous immunoglobulin; *PE* plasma exchange; *ICU* transfer to an intensive care unit) and results from anti-ganglioside antibody testing

### sNfL levels are higher in patients with GBS in comparison with controls

**Fig. 2:**
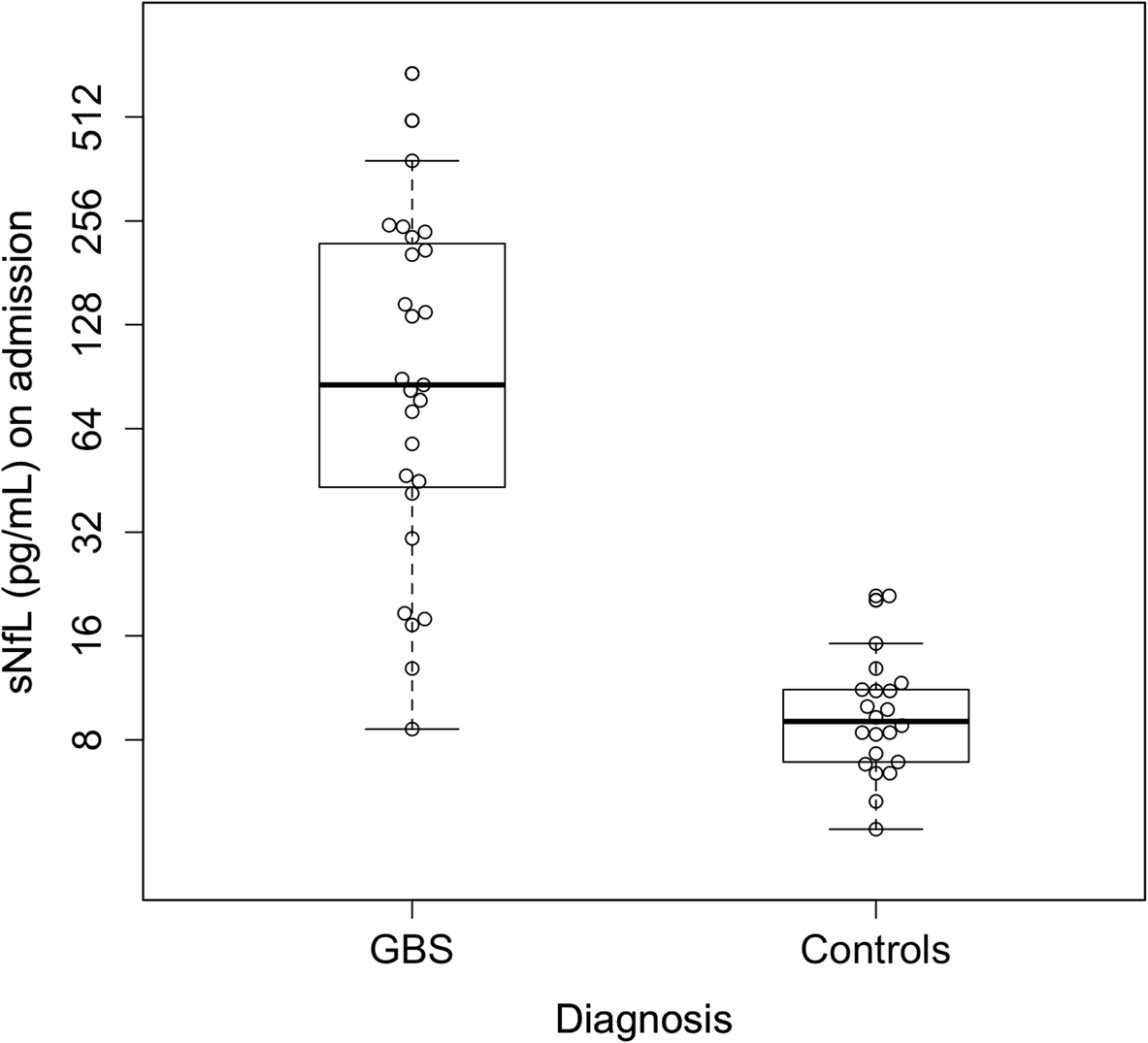
Serum neurofilament light chain (sNfL) concentrations upon admission in Guillain-Barré syndrome (GBS) patients and controls. Each dot represents a single individual. Box plots indicate median and IQR with whiskers extending 1.5 times the IQR

In order to investigate differences in sNfL levels in GBS patients compared with non-inflammatory controls, we calculated medians and means for both groups. The median (IQR) sNfL concentration on admission in the GBS group was 85.5 pg/ml (41.5–229.7 pg/ml) which distinguished them from controls that had a median concentration of 9.1 pg/ml (6.9–11.2 pg/ml, *t* test; *p* < 0.0001 (Fig. 2). CSF results from our control cohort suggested no presence of inflammatory activity based on cell count (2.0 [2–4]) and total protein (32.7 pg/ml [23.5–42.6 pg/ml]).

### sNfL in GBS show some correlation with conventional CSF parameters

Seventeen of 27 GBS patients showed albumin-cytological dissociation in the CSF, so we tested for an association between sNfL and conventional CSF parameters such as total protein and albumin index. The calculated medians (IQR) were 54.8 pg/ml (45.4–76.4 pg/ml) for CSF protein and 12.6 ([× 1000], 5.3–15.8) for the albumin index. Correlations of these parameters with sNfL were moderate in both cases (Spearman correlation coefficients *r*_*s*_ = 0.48; *p* = 0.016 for CSF protein and *r*_*s*_ = 0.57; *p* = 0.002 for albumin index), reflecting the increased permeability of the blood nerve and blood brain barrier and some transit of proteins between compartments.

### Consideration of age and sex in our study cohorts

A correlation of sNfL and age has been reported previously, and thus we tested for age as a confounder in both our study cohorts [29, 30]. The mean age in the GBS group was 55 years ± 19 (range 20–80 years) and 36 years ± 13 (range 18–60 years) in the control group. Analysis of covariance suggests no influence of age (*p* = 0.95) and sex (*p* = 0.60) on sNfL concentrations, whereas the age- and sex-adjusted group difference was preserved (*p* < 0.0001). In our control group, age and sNfL levels were clearly associated (*r*_*p*_ = 0.60, *p* = 0.0.004) which was not the case for our GBS patients (*r*_*p*_ = − 0.11, *p* = 0,587).

### Treatment strategies

Patients were treated with IVIg, PE or a combination of both. All 27 patients were treated with IVIg. Sixteen (59.3%) patients were treated with IVIg alone and nine patients (33.3%) underwent additional PE. Ten patients were transferred to an ICU, eight of them received IVIg and PE concomitantly, two of them did not undergo PE.

### sNfL levels correspond to neurological function

**Fig. 3:**
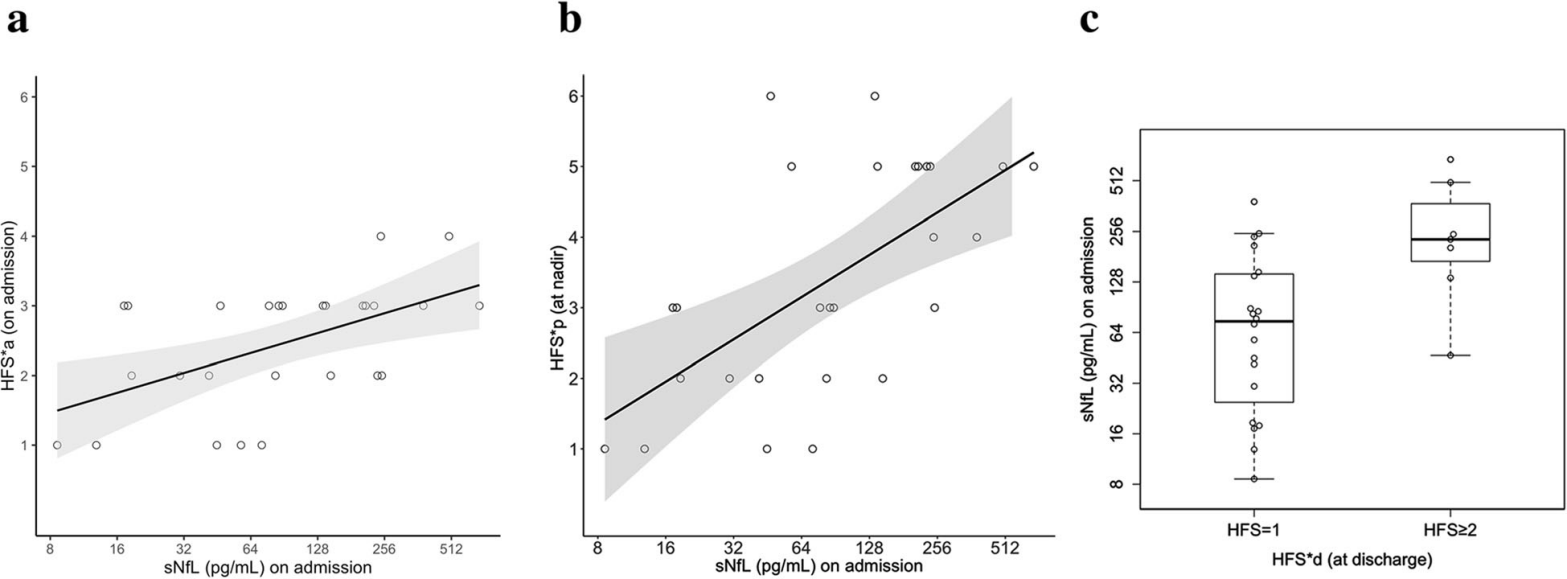
Correlation of serum neurofilament light chain (sNfL) concentrations upon admission with the Hughes Functional Score (HFS) calculated **a** on admission (HFS*a) with a Spearman’s correlation coefficient *r*_*s*_ of 0.52 (*p* = 0.005) and **b** at nadir (HFS*n) with an *r*_*s*_ of 0.59 (*p* = 0.001). Each dot in the scatter plot represents a sample, the line the estimated linear regression. **c** sNfL levels on admission in association with HFS at discharge (HFS*d) as a dichotomised outcome HFS = 1 and HFS ≥ 2. Each dot represents a single individual. Box plots indicate median and IQR with whiskers extending 1.5 times the IQR

In order to quantify neurological deficit in our GBS patients, HFS was calculated at admission (HFS*a), nadir (HFS*n) and hospital discharge (HFS*d) and related to sNfL levels upon admission. The median HFS values for these three time points were HFS*a = 3 (range 1–4), HFS*n = 3 (range 1–6) and HFS*d = 1 (range 1–6, Table 2). We found a moderate correlation between sNfL levels and HFS*a (Spearman correlation coefficient *r*_*s*_ = 0.52; *p* = 0.005; Fig. 3a), HFS*n (*r*_*s*_ = 0.59; *p* = 0.001; Fig. 3b) and HFS*d (*r*_*s*_ = 0.41; *p* = 0.04). As for the HFS at discharge, we dichotomised it into HFS = 1 and HFS ≥ 2 and found that sNfL levels at the time of clinical diagnosis were able to differentiate these groups (odds ratio [OR] per twofold increase, 2.71 [95%CI 1.10–6.67]; *p* = 0.03; Fig. 3c). Effects of sNfL on the outcome HFS*a were 0.29 ± 0.09 (*β* ± SE, *p* = 0.004) in the univariate (unadjusted) and 0.28 ± 0.09 (*β* ± SE, *p* = 0.006) in the multivariable (adjusted for age and preceding infection) linear regression model. For HFS*n, our analysis yielded a parameter estimate of 0.60 ± 0.15 (*β* ± SE, *p* = 0.0006) in the univariate (unadjusted) model and 0.60 ± 0.16 (*β* ± SE, *p* = 0.0008) in the multivariable (adjusted) model.

**Table 2:** Functional performance throughout the hospital stay

HFS	HFS*a *n* (%)	HFS*n *n* (%)	HFS*d *n* (%)
1	5 (19)	4 (15)	20 (74)
2	7 (26)	5 (19)	1 (4)
3	13 (48)	6 (22)	3 (11)
4	2 (7)	2 (7)	1 (4)
5	0 (0)	8 (30)	0 (0)
6	0 (0)	2 (7)	2 (7)

Distribution of the calculated values for the Hughes Functional Score (HFS) on admission (HFS*a) at nadir (HFS*n) and discharge (HFS*d)

*n* the absolute number

*%* the percentage

### sNfL levels on admission predict duration of hospitalisation and ICU transfer

**Fig. 4:**
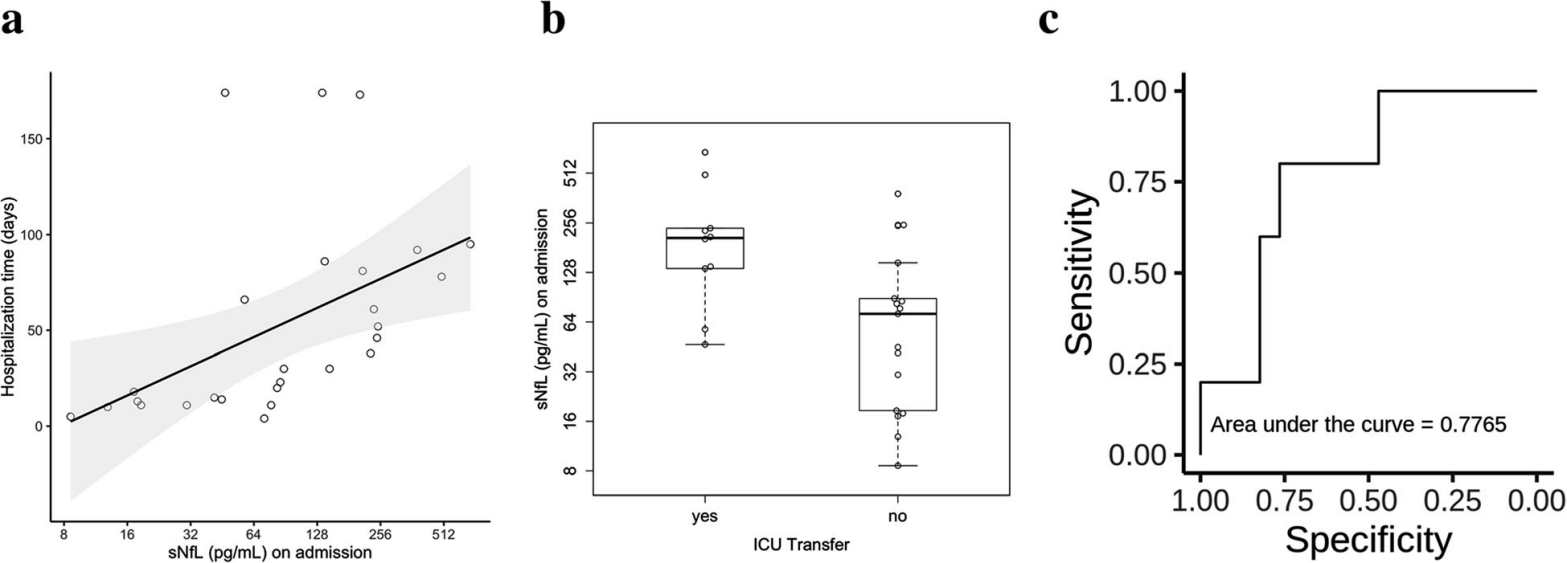
Correlation between serum neurofilament light chain (sNfL) concentrations upon admission. **a** The number of days hospitalised. Each dot in the scatter plot represents a sample, Spearman’s correlation coefficient *r*_*s*_ is 0.69 (*p* < 0.0001). **b** sNfL levels on admission in patients who did or did not require ICU (intensive care unit) transfer throughout their hospital stay. Box plots indicate median and IQR with whiskers extending 1.5 times the IQR. **c** Receiver operating characteristic (ROC) curve analysis for the probability of ICU transfer depending on sNfL levels on admission

We evaluated the duration of hospitalisation and indications for referrals to an intensive care unit. The median number of hospitalised days was 30 (range 13‑66 days) in the GBS group. Higher sNfL levels on admission correlated with a longer hospitalisation time (*r*_*s*_ = 0.69; *p* < 0.0001 Fig. 4a). Cox regression estimated a hazard ratio (HR) of 0.62 (95% CI 0.46–0.85; *p* = 0.003), revealing a lower chance of early discharge from the hospital with higher sNfL levels. This relation was independent of age and a preceding infection, with an adjusted HR of 0.60 (95% CI 0.44–0.82; *p* = 0.001). Ten of 27 patients were transferred to an ICU. The likelihood of this intervention was associated with raised sNfL concentration on admission with an odds ratio of 2.37 (95% CI 1.14–4.95 Fig. 4b). The prognostic importance was evaluated performing a ROC (receiver operating characteristic) curve analysis and revealed an AUROC (area under the ROC curve) of 0.78 (95% CI 0.60–0.96 Fig. 4c).

### sNfL levels deviating from the median illustrate a different outcome

In a different approach to prognosis, we calculated patient outcome for baseline sNfL levels below and above the median. As described before, the median sNfL concentration in our sample was at 85.5 pg/ml. For the investigation of potential differences in outcome above and below this threshold, we analysed the frequency distribution of the following parameters: *hospitalisation days*, *HFS* and *ICU transfer*. Patients with sNfL levels below the median had a median hospital stay of 13.5 days, versus 78 days in the group with sNfL above the median. The median HFS score at nadir was 2 (=able to walk at least 5 m) for patients below that threshold and 5 (=ventilated) for patients above that threshold. Correspondingly, patients with a baseline sNfL concentration above the median in our sample were transferred to an ICU more frequently (62%) than patients below the median (14%). Results from this descriptive analysis are given in Table 3.

**Table 3:** Patient outcomes for baseline sNfL levels below and above the median

	sNfL ≤ 85.5 pg/ml	sNfL > 85.5 pg/ml
	*n* = 14	*n* = 13
Hospitalisation days†	13.5 (11–20)	78 (46–92)
HFS*a†	2 (1–3)	3 (3–3)
HFS*n†	2 (1–3)	5 (4–5)
HFS*d†	1 (1–1)	1 (1–3)
HFS*d = 1‡	13 (93%)	7 (54%)
ICU transfer‡: *yes*	2 (14%)	8 (62%)
ICU transfer‡: *no*	12 (86%)	5 (38%)

Descriptive analysis of different outcome parameters below and above the median serum neurofilament light chain (sNfL) concentration upon admission

HFS*a Hughes Functional Score on admission, HFS*n Hughes Functional Score at nadir, HFS*d Hughes Functional Score at discharge, *ICU* intensive care unit

†median (IQR)

‡*n* (%)

## Discussion

Although the diagnosis of Guillain-Barré syndrome is well established by means of clinical criteria with supportive electrophysiology and CSF investigations, a convenient and reliable biomarker for the prediction of clinical outcome or prognosis is still needed. In current literature, the potential of NfL as a biomarker in a variety of neurological diseases including neuropathies is being discussed. A recent study based on a sample of 25 patients with acquired neuropathies, including five cases of GBS, suggested NfL as a potential biomarker in correlation with the patients’ disability [24]. Another study with 18 patients showed that high NfL concentrations in *cerebrospinal fluid* at onset of GBS may predict long-term disability, thus, reflecting affirmatively on the mechanisms of axonal damage in this disease [21]. We think our present investigation of 27 patients, though different in design and hypotheses, compares well to this observation by suggesting NfL concentrations in *serum* to be linked with neurological impairment or burden of hospitalisation.

We demonstrated that sNfL levels, obtained upon admission, were increased compared with controls. In addition, we found that sNfL levels are clearly correlated with parameters for disease severity and indicative of the duration of hospitalisation and the risk of transfer to an ICU. With regard to functional outcome, we found in univariate (unadjusted) linear regression models that a twofold higher sNfL concentration upon admission resulted in an HFS-increase of 0.3 on admission and 0.6 at nadir. This was confirmed in the multivariable (adjusted) linear regression models. As for the HFS on discharge, our results indicate that doubling the sNfL concentration upon admission makes it almost three times more likely to have a less favourable outcome (HFS ≥ 2). Furthermore, higher sNfL levels on admission correlated strongly with duration of hospitalisation (*r*_*s*_ = 0.69, *p* < 0.0001). Our calculations estimated an HR per twofold increase of 0.62, implying that the likelihood of discharge is reduced to 62% when the sNfL concentration on admission doubles. Respectively, baseline sNfL levels were associated with the need of intensive care: ICU transfer occurred at an OR per twofold increase of 2.37, showing that transfer to ICU was about 2.4 times more likely when the sNfL concentration on admission doubles. The median sNfL concentration on admission was 85.5 pg/ml, a value well in line with another study that reported a median of 84.7 pg/ml in acute neuropathies including five cases of GBS [24]. In order to illustrate the potential of sNfL as an indicator for disease course, we calculated our defined outcome parameters (hospitalisation time, Hughes Functional Score, ICU transfer) for a threshold of below and above the median. By means of this model, descriptive analyses demonstrated that the median number of days spent in the hospital were six times higher above that set threshold. The chance of being discharged with an unimpaired walking ability was 93% for patients with sNfL levels below 85 pg/ml upon admission. Also, the likelihood of complications leading to ICU transfer was only 14%.

There are some limitations of our study. We were constrained to relating our data exclusively to patient records evaluated retrospectively and to a single biomarker measure upon admission. However, accuracy of diagnosis was ascertained by applying selective screening criteria. Our controls were carefully selected to reflect conditions without CSF inflammation or likely axonal damage. Though our studied GBS cohort is indeed the largest one to date, the sample size is small. In our tertiary care hospital we see about eight to ten cases of GBS per year which we regard as a representative patient collective for Austria with a reported incidence of GBS of 1/100,000 [31, 32]. Our cohort consisted of 27 participants. Yet, in comparison with other studies on both blood and CSF derived neurofilaments in GBS (two studies on NfH and one on NfL) with sample sizes ranging from 3 to 18, our number of enrolled patients suggests reasonable validity [21, 23, 24]. Also, our predictive model did not aim at giving a strict cut-off. We rather intended to approximate an idea of a range of sNfL concentration that indicates a shift in patient outcomes and we were able to show how patient outcomes worsened with increasing sNfL concentrations. In addition, 10 of 27 patients were transferred to an ICU, a number slightly higher than previously reported [2, 33]. Eight of these transferred patients underwent PE (vs. one patient receiving PE on regular care). It would be interesting to adjust for this possibly confounding factor; yet, due to our small sample size and the retrospective design of this study, we cannot fully distinguish the reasons for this observation. In any case, clinical status was attributable in this regard: The patient’s worst clinical status (as indicated by the outcome HFS*p) seemed different in those patients transferred to ICU. Their median (IQR) HFS*p scores were 5 [5] vs. 2 [2, 3] in those not transferred. Procedural considerations may also have played a role, as patients undergoing PE were, apparently, more likely to be moved to intensive care as opposed to patients treated with immunoglobulin.

The findings from our study are relatable to current literature and reveal new aspects with respect to sNfL as a biomarker in GBS [13, 32, 34]. Interestingly, age did impact sNfL concentrations in our control group but not in our patient cohort. Control patients showed a clear association between sNfL and age (*r*_*p*_ = 0.60, *p* = 0.0.004), whereas the GBS group did not (*r*_*p*_ = 0.11, *p* = 0.587). This is very likely to be due to the significant increase in NFL levels and decreased integrity of the blood nerve and blood brain barrier which supervenes any normal physiological protein distribution. We also found that sNfL concentrations did not seem to differ between the subtypes of AIDP and axonal variants which are somewhat curious. Though sample size is too small to draw any conclusions, we believe that sNfL are elevated even in primarily demyelinating disease which might be attributed to axonal damage below the threshold detectable by nerve electrophysiology. Neurophysiology may not represent what is really happening at the pathology level. Nodoparanodopathy has been described as an emerging concept of damaged peripheral nerves [35]. Neurofilaments may be leaking from many fibres that are being damaged long before electrophysiology is capable of determining an excess of axonal loss in NCS or electromyography. In a prospective study where we are able to look at eventual outcomes by late NCS or eventual disability outcomes it may be possible to discern this in a larger study.

In total, we were able to further add to findings from previous studies with respect to a more profound representation of the patients’ in-hospital disease course including rigorous evaluation of the diagnosis. We believe our results are of mediate relevance to caregivers and patients alike. With our data at hand, NfL levels detected early in serum of patients with GBS could enable individualised risk stratification and prognosis in the future. They may be synergistically useful when included in other clinical predictive models, such as the Erasmus GBS outcome scale (mEGOS) [12].

## Conclusion

In this retrospective analysis, we show multifaceted associations between serum neurofilament light chain concentrations and outcomes in Guillain-Barré syndrome. This is the largest and most comprehensive study of NFL to date. We systematically analysed records of 27 GBS patients, compared them to well-defined controls and exposed correlations between sNfL and clinical outcome. Our predictive model revealed that patients with baseline levels below our sample’s median of 85 pg/ml had a 93% chance of being discharged with an unimpaired walking ability. Also, we found clear associations with hospitalisation days and the probability for ICU transfer.

We believe that the knowledge of this correlation will influence prognostic considerations in this rare disease. Not only could this spur further research endeavours targeting this biomarker’s role in GBS but it would also aid in the process of identifying patients at higher risk of poor outcome, thus, eventually resulting in a more aggressive therapeutic approach to these particular patients. We have initiated a prospective study with collaborators that shall validate this biomarker further. By relating to a longitudinal follow up that also accounts for quality of life of those affected, we believe that patient care can be improved in the future by recognising those at higher risk.

## Data Availability

Data can be made available from the corresponding author upon reasonable request and after approval from the ethics review board at the Medical University of Vienna.

## Abbreviations

AIDP: Acute inflammatory demyelinating polyradiculoneuropathy
AMAN: Acute motor axonal neuropathy
AMSAN: Acute motor and sensory axonal neuropathy
AUC: Area under the curve
CI: Confidence interval
CNS: Central nervous system
CSF: Cerebrospinal fluid
HFS: Hughes Functional Score
HFS*a: Hughes Functional Score at admission
HFS*d: Hughes Functional Score at discharge
HFS*n: Hughes Functional Score at nadir
HR: Hazard ratio
ICU: Intensive care unit
IQR: Interquartile range
IVIg: Intravenous immunoglobulin
NfH: Neurofilament heavy chain
NfL: Neurofilament light chain
OR: Odds ratio
PE: Plasma exchange
pg/ml: Pictogram per millilitre
ROC: Receiver operating characteristic
SD: Standard deviation
Simoa: Single molecular array
sNfL: Serum neurofilament light chains
